# SGLT2 inhibitor as a potential therapeutic approach in hyperthyroidism-induced cardiopulmonary injury in rats

**DOI:** 10.1007/s00424-024-02967-4

**Published:** 2024-05-03

**Authors:** Nermeen Bastawy, Aliaa E. M. K. El-Mosallamy, Samira H. Aljuaydi, Huda O. AbuBakr, Rabab Ahmed Rasheed, A. S. Sadek, R. T. Khattab, Wael Botros Abualyamin, Shereen E. Abdelaal, Amy F. Boushra

**Affiliations:** 1 Department of Medical Physiology, Faculty of Medicine, Cairo University, Cairo, Egypt; 2 Department of Pharmacology, Medical Research and Clinical Studies Institute, National Research Centre, Giza, Egypt; 3 Department of Biochemistry and Molecular Biology, Faculty of Veterinary Medicine, Cairo University, Giza, 12211 Egypt; 4 Department of Medical Histology and Cell Biology, Faculty of Medicine, King Salman International University, El Tor, 46511 South Sinai Egypt; 5 Department of Anatomy and Embryology, Faculty of Medicine, Ain Shams University, Cairo, 11566 Egypt; 6 Department of Anatomy and Embryology, Faculty of Medicine, King Salman International University, El Tor, 46511 South Sinai Egypt; 7 Department of Medical Physiology, Faculty of Medicine, Fayoum University, Fayoum, Egypt; 8 Department of Natural and Physical Sciences, Blinn College, Brenham, TX USA; 9 Department of Pathology, Medical Research and Clinical Studies Institute, National Research Centre, Giza, Egypt; 10 Department of Biochemistry, Faculty of Veterinary Medicine, Egyptian Chinese University, Cairo, Egypt

**Keywords:** Dapagliflozin, Hyperthyroidism, Cardiopulmonary, Toxicity, Antioxidant, TNF-α

## Abstract

Hyperthyroidism-induced cardiac disease is an evolving health, economic, and social problem affecting well-being. Sodium-glucose cotransporter protein 2 inhibitors (SGLT2-I) have been proven to be cardio-protective when administered in cases of heart failure. This study intended to investigate the potential therapeutic effect of SGLT2-I on hyperthyroidism-related cardiopulmonary injury, targeting the possible underlying mechanisms. The impact of the SGLT2-I, dapagliflozin (DAPA), (1 mg/kg/day, p.o) on LT4 (0.3 mg/kg/day, i.p)-induced cardiopulmonary injury was investigated in rats. The body weight, ECG, and serum hormones were evaluated. Also, redox balance, DNA fragmentation, inflammatory cytokines, and PCR quantification in heart and lung tissues were employed to investigate the effect of DAPA in experimentally induced hyperthyroid rats along with histological and immunohistochemical examination. Coadministration of DAPA with LT4 effectively restored all serum biomarkers to nearly average levels, improved ECG findings, and reinstated the redox balance. Also, DAPA could improve DNA fragmentation, elevate mtTFA, and lessen TNF-α and IGF-1 gene expression in both organs of treated animals. Furthermore, DAPA markedly improved the necro-inflammatory and fibrotic cardiopulmonary histological alterations and reduced the tissue immunohistochemical expression of TNF-α and caspase-3. Although further clinical and deep molecular studies are required before transposing to humans, our study emphasized DAPA’s potential to relieve hyperthyroidism-induced cardiopulmonary injury in rats through its antioxidant, anti-inflammatory, and anti-apoptotic effects, as well as via antagonizing the sympathetic over activity.

## Introduction

The primary active type of thyroid hormones, T3, is well known for regulating growth, metabolism, and a host of other physiological processes [66]. Elevated thyroid hormone levels are a sign of hyperthyroidism, a clinical condition involving thyroid malfunction. In nations where iodine is sufficient, its prevalence ranges from 0.2 to 1.3% [15]. Most of the effects of thyroid hormone are mediated by nuclear receptors and affect the transcription of T3-responsive genes. Significant evidence, however, suggests that some dysfunctions brought on by hyperthyroidism are caused by tissue oxidative stress [6]. Numerous tissues, including blood vessels and myocardium, contain thyroid hormone receptors. Therefore, variations in thyroid hormone concentrations may impact cardiovascular functions [54].

Since the turn of the 20th, extensive research has been done on the impact of thyroid dysfunction on the cardiovascular system. Clinically, thyroid hormone excess or deficiency may cause or worsen cardiovascular disease (CVD) [12]. The prevalence of hyperthyroidism-related cardiac problems appears to be higher in older individuals with Graves’ disease or toxic untreated hyperthyroidism [40]. Hyperthyroidism may affect functional parameters of the left ventricle, including the ejection fraction [67]. About 6% of hyperthyroid patients appear to have heart failure as their most common clinical presentation, with roughly half of them having left ventricular systolic dysfunction. Compared to euthyroid subjects, patients with symptomatic heart failure and an ejection fraction < 35% have an approximately 85% higher relative risk of dying [46, 58]. Moreover, systolic blood hypertension affects up to 68% of hyperthyroid patients, particularly the older age group [42].

There is a correlation between tissue oxidative injury and increased thyroid hormone levels in the blood. Most of the data suggests that an experimentally induced hyperthyroid state causes oxidative damage to many tissues, including the heart and lungs, changing the pro-oxidant-antioxidant balance characteristics of euthyroid tissues [59]. Increases in lung cell damage, the permeability of the alveolocapillary membrane, the total number of phagocytic cells, and the phagocytic cells’ enhanced release of nitric oxide were all caused by hyperthyroidism [75]. Moreover, increased thyroid hormone levels can boost lung responses to other substances that cause oxidant stress, as evidenced by the increased pulmonary toxicity of oxidant gases in hyperthyroidism [25].

Sodium-glucose cotransporter protein 2 inhibitors (SGLT2-I) are oral anti-diabetic medications that lower blood sugar by lessening the kidney’s proximal convoluted tubules’ renal glucose reuptake [27]. Within the past few years, many studies researched the cardioprotective role of SGLT2 inhibitors, such as empagliflozin, dapagliflozin, and canagliflozin, in heart failure [53, 80]. Interestingly, the cardiovascular profits of SGLT2-I result from mechanisms other than glycemic control. Moreover, SGLT2-I cardioprotective effects were exhibited even in non-diabetic patients with established CVD [73]. SGLT2-I reduces oxidative stress by translocating Nrf2 to the nucleus and triggering Nrf2/ARE signaling, which lessens myocardial fibrosis by inhibiting collagen deposition through the traditional TGF-β/Smad pathway [41].

With a focus on potential mechanisms by which SGLT2-I may exert their therapeutic effects, this study points to the potential therapeutic impact of dapagliflozin on rats’ cardiopulmonary injury caused by hyperthyroidism and highlights the possible underlying mechanisms.

## Materials and methods

### Drugs

Levothyroxine sodium hydrate (LT4) (powder form, pack size: 50 mg, Sigma-Aldrich, MO, USA) and dapagliflozin (DAPA) (5 mg film-coated tablet, AstraZeneca Pharmaceuticals LP, Indiana, USA) were used in this study. Both drugs were of analytical grade and were dissolved in normal saline 0.9% just prior to use.

### Animals housing and ethical statement

Twenty-four adult male albino rats of Wistar strain (10–12 weeks, 180–200 g) were purchased and sheltered in the Animal House of the Faculty of Medicine, Cairo University, Egypt. Per the NIH Guidelines for the Care and Use of Laboratory animals and strictly adherent to the ARRIVE guidelines (Animal Research: Reporting In Vivo Experiments), animals were kept in a hygienic, naturally ventilated, and pathogen-free environment in conventional stainless-steel cages (6 rats/cage). Rats were permitted to acclimate before starting the study for 1 week with unrestricted access to rat chow and tap water at a temperature oscillating between 22:24 °C, moderate humidity (50–60%), and alternating 12/12-h dark/light cycle. This study was accepted by The Institutional Animal Care and Use Committee, Cairo University (CU-IACUC), Egypt (Code: CU/III/F/15/23).

### Sample size calculation

The number of animals per group was calculated by employing the method described by [68] before conducting the study to minimize the number of used animals. As per previous work [60], we considered the mean difference in serum T4 level between the study groups to be 5.65, and the standard deviation was 1.46. Thus, the sample size is estimated as six rats per group, with a total of 24 rats attaining a power of 80% when *p* value is adjusted at 0.05 or less as a level of significance.

### Experimental design

**Fig. 1:**
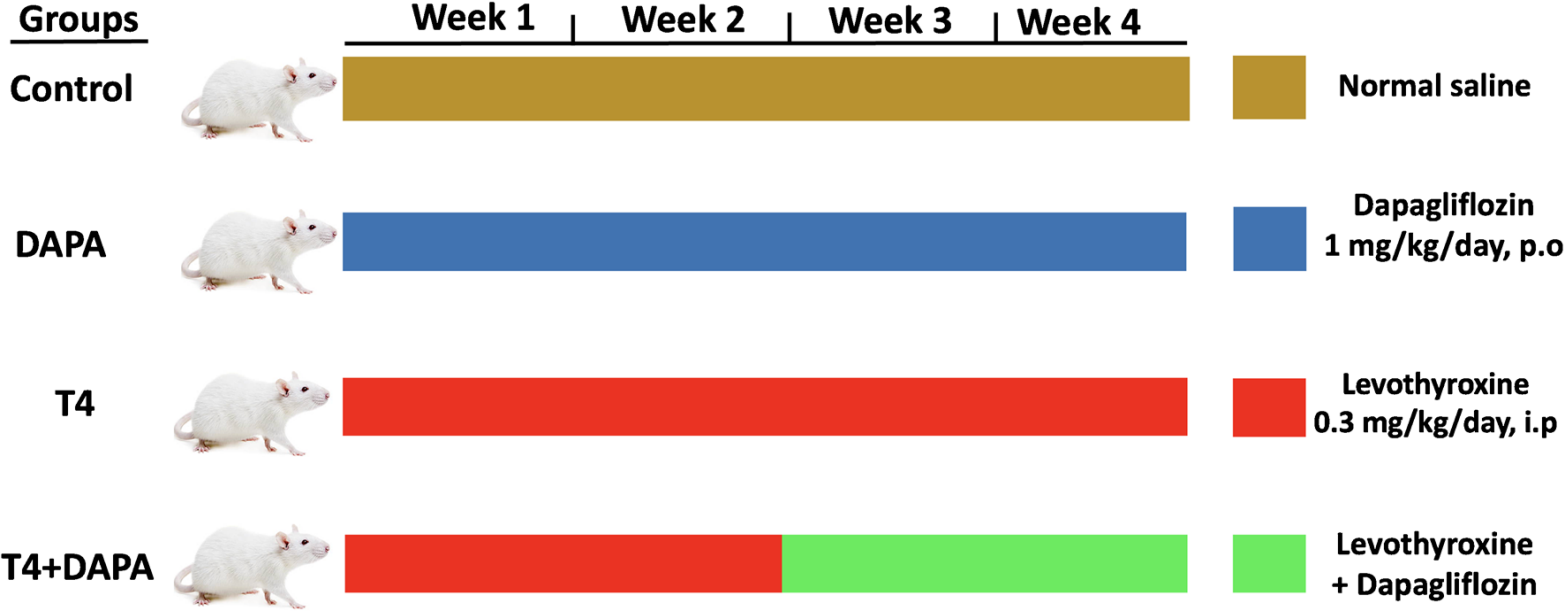
The timeframe, drug dosages, routes, and animal grouping

The rat model of thyrotoxicosis was built up by daily intraperitoneal (i.p) injection of levothyroxine (LT4) at a dose of 0.3 mg/kg dissolved in saline for 4 weeks [37]. Rats were equally and undiscriminatingly assigned to four groups (*n* = 6). For randomization of the rats, they were labeled from 1 to 24 and were allocated into four groups using the Rand function in Excel (ver. 16.82). The animal groups were as follows: the control group: received the saline vehicle (1 mL/kg/day, p.o) for 4 weeks; DAPA group: received DAPA dissolved in normal saline (1 mg/kg/day, p.o) for 4 weeks [52], T4 group: received LT4 (0.3 mg/kg/day, i.p) for 4 weeks, and T4 + DAPA group: received LT4 solely for 2 weeks, then received both LT4 and DAPA for a further 2 weeks at the previously assigned doses and routes for each drug. The timeframe, drug dosages, routes, and animal grouping are shown in Fig. 1.

At the end of the fourth week, rats were weighed, fasted overnight (12 h), anesthetized by ketamine injection (100 mg/kg, i.p) [34], and then, prepared for ECG monitoring, blood, and sample collection.

### Measurement of body weight

The rats’ body weight was recorded using a digital scale (PS 750.R1) starting from day zero before administering any medications and continued weekly till the end of the study (day 28).

### ECG and heart rate recording

After the conclusion of the trial, the anesthetized rats were put supine on a wooden sterilized board for ECG monitoring. Briefly, needle electrodes were implanted subcutaneously in the right and left paws (front and back sides) of each animal for five minutes. Being connected to PowerLab 8SP (model ML785), Lead II ECG signals and the heart rate were monitored, recorded, and analyzed using ECG analysis version 2.0 software (AD instruments) [20].

### Blood collection

After ECG monitoring, retro-orbital blood samples (3 mL) were collected from each anesthetized rat into evacuated tubes. The serum was then separated by centrifugation (3000 rpm for 10 min at 4 °C). The collected serum was stored at − 20 °C before biochemical lab investigations.

### Tissue collection

A thoracotomy incision was immediately done on the anesthetized rats to extract the heart and lungs, followed by washing with cold saline. The left ventricle was dissected into two halves. Half the left ventricle and the right lung were sent for histopathological examination. The other half of the left ventricle and the left lung were divided into two sets. The first set was homogenized in cold PBS (pH 7.4) using a Teflon homogenizer, and the homogenates were centrifuged at 14,000 × *g* for 20 min at 4 °C. The obtained supernatant was used to assess oxidant and antioxidant tissue biomarkers. The other set of samples was kept frozen immediately in liquid nitrogen at − 80 °C until the processing for RT-qPCR analysis.

### Biochemical analysis

Serum levels of thyroid-stimulating hormone (TSH), T3, and T4 were analyzed by colorimetric competitive enzyme immunoassay employing individual ELISA kits (Abcam, United States), according to the manufacturer’s protocol. Also, lactate dehydrogenase (LDH) (LDHI 0108021) and creatine kinase-myocardial band (CK-MB) (CKMB0101022-3) by using kits are supplied by Spectrum diagnostics and measured using the NS BIOTEC Chemistry analyzer (MODEL: SCA-1200).

### Oxidant/antioxidant tissue biomarkers

#### Assessment of catalase activity

According to [3], CAT activity was tested using Biodiagnostic kits (Egypt). A catalase inhibitor was used to halt the reaction of catalase with a known amount of H2O2 after 1 min. The remaining H2O2 combines with 3–5-Dichloro-2-hydroxybenzene sulfonic acid and 4-aminophenazone in the presence of peroxidase to generate a chromophore whose color intensity is inversely proportional to the concentration of catalase in the sample. The UNICO-UV-2100 spectrophotometer was used to measure the absorbance at 510 nm.

#### Assessment of reduced glutathione

According to [19], the reduced glutathione (GSH) level was evaluated using Biodiagnostic kits (Egypt). The tissue homogenate was combined with 0.2 M phosphate buffer pH 8 and 5, 50-dithiobis 2-nitrobenzoic acid (DTNB). The reduction of DTNB is a prerequisite for the decline of glutathione levels. Glutathione gives off a yellow color, and the UNICO-UV-2100 spectrophotometer detected its absorbance at 412 nm.

#### Assessment of lipid peroxidation

According to [48], the concentration of malondialdehyde (MDA) was utilized as a measure of lipid peroxidation. MDA was measured using Biodiagnostic kits (Egypt) via evaluating the reactive species of thiobarbituric acid [74]. MDA was established using a UNICO-UV-2100 spectrophotometer; the absorbance of the resulting pink product was measured at 534 nm.

#### Assessment of genotoxicity by DNA fragmentation

The DNA fragmentation percentage was calculated using the procedure that was provided by [1]. In summary, 400 μL of hypotonic lysis buffer containing 10–20 mg tissue was mixed and centrifuged at 3000 × *g* for 15 min at 4 °C. The supernatant was separated into two portions: one was used for the gel electrophoresis, and the other was combined with the pellet to measure the amount of fragmented DNA by the diphenylamine using the UNICO-UV-2100 spectrophotometer at 578 nm. DNA fragmentation percentage in each sample was expressed by the formula:

$$\%\mathrm D\mathrm N\mathrm A\;\mathrm f\mathrm r\mathrm a\mathrm g\mathrm m\mathrm e\mathrm n\mathrm t\mathrm a\mathrm t\mathrm i\mathrm o\mathrm n=(\text{O}.\mathrm D\;\mathrm{Supernatant}/\text{O}.\mathrm D\;\mathrm{Supernatant}\;+\;\mathrm O.\mathrm D\;\mathrm{Pellet})\;\times\;100$$

### Quantitative real-time polymerase chain reaction of mitochondrial transcription factor A (mtTFA), tumor necrosis factor-alpha (TNF-α), and insulin-like growth factor-1 (IGF-1) genes

RNA was extracted from heart and lung tissues using the QIAmp miRNAsy mini kit (QIAGEN, Hilden, Germany) as directed by the manufacturer. The purity and concentration of total RNA were determined using a nanodrop ND-1000 spectrophotometer. Reverse transcriptase was utilized to synthesize cDNA from the extracted RNA (Fermentas, EU). Using a mixture of 1 μL cDNA, 0.5 mM of each primer (Table 1), and iQ SYBR Green Premix (Bio-Rad 170–880, USA), real-time PCR (qPCR) was carried out in a total volume of 20 μL. The MyiQ real-time PCR detection device and the Bio-Rad iCycler heat cycler were used to accomplish PCR amplification and analysis. Every assay comprises three duplicate samples of each cDNA under test along with a negative control that has no template; the expression in relation to the control is determined using Eq. 2^−ΔΔCT^ [2].

**Table 1:** Primer sequences of reference, mtTFA, TNF-α, and IGF-1 genes of *Rattus norvegicus*

Target genes	Accession no	Sequence (5′ to 3′)	Product size
GAPDH (reference gene)	NM_017008.4	F: 5′-GAGACAGCCGCATCTTCTTG-3′ R: 5'- TGACTGTGCCGTTGAACTTG -3'	224 bp
mtTFA	NM_031326.2	F: 5′-CATGACGAGTTCTGCCGTTTG-3′ R: 5′-AGTAAAGCCCGGAAGGTTCTT-3′	254 bp
TNF-α	NM_012675.3	F:5′-ACACACGAGACGCTGAAGTA-3′ R:5′-GGAACAGTCTGGGAAGCTCT-3′	235 bp
IGF-1	XM_039078402.1	F: 5′-TCTCCTAGTCCCTGCCTCTT-3′ R: 5′-TCTGTGAAGGAAGCGGCTTA-3′	183 bp

### Histological examination

Fresh portions from heart and lung tissues were dissected, instantly fixed in 10% neutral buffered formalin for 24 h, dehydrated in scaling grades of alcohol, cleared in xylene, and then, dipped in molten paraffin to obtain tissue blocks. Thin Sects. (3–4 μm) were sliced by a microtome, further stained with hematoxylin and eosin to assess the cardiopulmonary histopathological changes and picrosirius red stain to demonstrate the interstitial myocardial and pulmonary fibrosis [71].

### Immunohistochemical analysis

As described by [56], thin Sects. (3–4 micron) were cut from the paraffin blocks on positively charged slides. Following deparaffinization, the sections were treated with 0.3% H_2_O_2_/methanol and left for ten min, at room temperature to erase the action of peroxidase. Antigen retrieval was done in citrate buffer (vol. 10 mM, 95 °C, pH 6) and left to cool (1 h at room temperature). Sections were then incubated with primary antibodies overnight at 4 °C; the product codes and the source are as specified in Table 2. Later, sections were incubated in DAB to reveal peroxidase activity and left overnight in phosphate-buffered saline (PBS) at 4 °C. The procedure was finalized by adding DAB, followed by counterstaining with hematoxylin. Sections were examined for the brown cytoplasmic and nuclear areas, indicating a positive reaction against the blue background.

**Table 2:** Primary antibodies used for immunohistochemistry

Antibody	Caspase-3	TNF-α
Source	Diagnostic BioSystems, CA, USA	Elabscience, TX, USA
Catalog #	PDR172	E-AB-22159
Host/type	Rabbit, polyclonal	Mouse, monoclonal
Optimal dilution	Ready to use	1:200
Cellular localization	Cytoplasmic, some nuclear	Cytoplasmic
Function	Detect apoptosis	Detect inflammation and macrophage activity

### Image acquisition, morphometric measurements, and image analysis

**Fig. 2:**
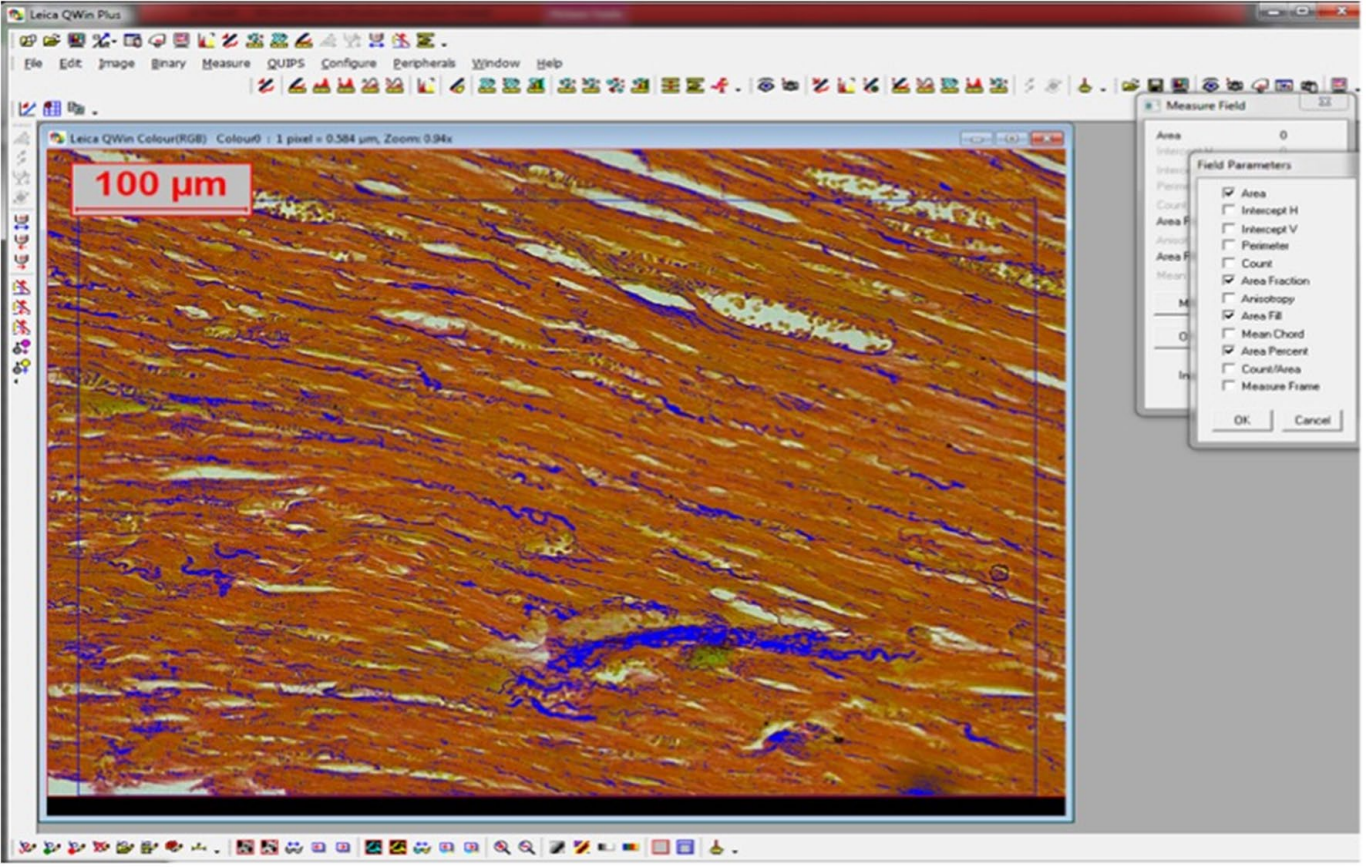
A binary image demonstrating the morphometric measurement of the area parameters (area, area fill, area fraction, and area percent) to detect the red-stained fibrous strand in the heart tissue (Picrosirius red, × 200)

H&E, picrosirius red, and immunohistochemically stained sections were examined and snapped at the Pathology & Image Analyzer Research Lab, Medical Research Centre of Excellence (MRCE), National Research Centre, Cairo, using the image analyzer Leica Qwin 500 (LEICA Image Systems Ltd., Cambridge, England). It comprised a Leica DM3000 microscope with a JVC color video camera attached to a computer system. To avoid bias in reporting the results, we used the “comprehensive masking method” according to [23]. Briefly, the tissue sections from different study groups were labeled as a, b, c, d, etc. They were examined by two contributors (a histologist and a pathologist) who were unaware of the study design or the treatment given to rats. Also, blinding was applied while recording ECG, biochemical measurements, and statistics. The morphometric measurements, taken from ten randomly chosen non-intersecting fields × 200, were carried out on H&E-stained sections of the heart to measure the wall thickness of the left ventricle, picrosirius red-stained sections to detect the percentage of the red-colored fibrous tissue, and immunohistochemically stained slides (for both Caspase-3 and TNF-α antibodies) to measure the percentage of positive cells. The area, area fraction, area fill, and area percentage were calculated using the automated system software. The areas to be measured were marked by a binary color to form a binary image (Fig. 2). The area of positivity was determined as an area in micrometers squared per field. The results appeared automatically on the monitor as a table with the measured total, mean, standard deviation, standard error, minimum, and maximum area.

### Statistical analysis

The acquired values are given as means ± S.E of the mean. Comparisons of weight values in relation to interval weeks between different groups were performed by two-way analysis of variance (ANOVA) using SPSS software version 24. In other parameters, the comparisons between groups were carried out by one-way analysis of variance (ANOVA) followed by “Duncan’s Multiple Range” test for post hoc analysis using SPSS software version 24. The significance was set at *p* ≤ 0.05. GraphPad software Instat (version 2) was used to make graphs.

## Results

### Effect of DAPA on body weight in hyperthyroid rats

**Fig. 3:**
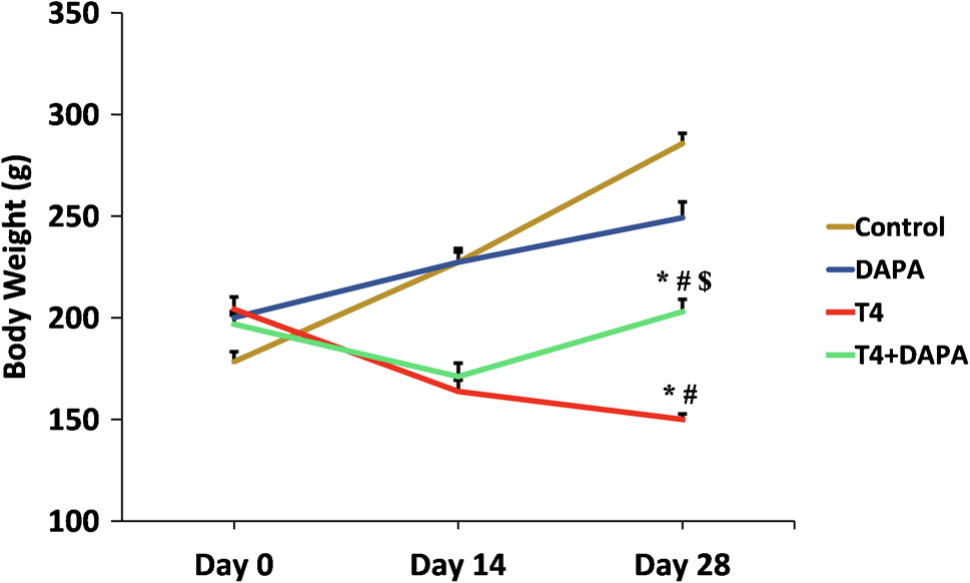
Weights in grams (g) in interval weeks of different groups. Values are mean ± SE, *n* = 6. *, #, and $ are significant variances from control, DAPA, and T4 groups in order at *p* ≤ 0.05

As shown in Fig. 3, there was a weight loss in the hyperthyroid rats (both untreated or treated with DAPA) starting after the first week of induction of hyperthyroidism and reaching significant values from the third week till the end of the experiment, compared to the control and DAPA groups (*p* ≤ 0.05). The concomitant treatment of the hyperthyroid rats with DAPA significantly ameliorated the weight loss compared to the T4 group (*p* ≤ 0.05). DAPA alone did not affect the body weight compared to the control group.

### Effect of DAPA on ECG in hyperthyroid rats

**Fig. 4:**
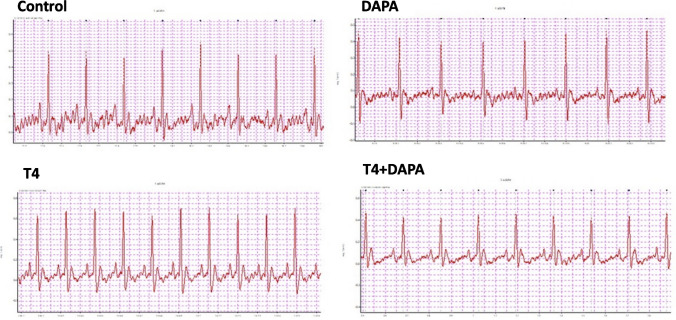
The ECG findings in the four studied groups, hyperthyroidism (T4) group showing significant increase in heart rate, shortened PR interval, with significant prolongation of QTc and JT intervals, as well as increased amplitude and duration of T wave. These changes are not detected in the recordings of hyperthyroid animals treated with DAPA

The results of the current study show a significant increase in heart rate in the animals with hyperthyroidism compared to the controls (*p* ≤ 0.05). Animals with hyperthyroidism treated with DAPA showed significantly lower heart rate levels as compared to the untreated animals (*p* ≤ 0.05). Additionally, the untreated hyperthyroid group showed significant prolongation in the JT interval compared to the control groups (*p* ≤ 0.05). This effect was ameliorated with DAPA treatment, with no significant difference from control levels, *p* = 0.7, 0.8). DAPA-treated group showed significantly shorter JT intervals as compared with the untreated group (*p* ≤ 0.05). ECG records from T4 group showed impaired repolarization of ventricles as indicated by the prolonged QT, *p* = 0.002, QTc duration, *p* ≤ 0.05), T wave abnormalities (prolonged T peak-T end, *p* ≤ 0.05) and T wave amplitude (*p* ≤ 0.05) as compared with the control group. Ventricular repolarization was significantly improved after DAPA treatment, as indicated by the significant difference in QT, QTc, and T peak-T end. T wave amplitude (*p* ≤ 0.05) from the hyperthyroidism group and the non-significant difference from the control groups (*p* = 0,7. 0.1, 0.7, and 0.5, respectively) (Table 3 and Fig. 4).

**Table 3:** The mean ± SE of the measured ECG parameters

Groups	Control	DAPA	T4	T4 + DAPA
HR (bpm)	260.2 ± 10.71	267.4 ± 8.57	383.4 ± 10.03*^#^	329.4 ± 9.06*^#$^
P amplitude (mv)	0.05 ± 0.01	0.04 ± 0.006	0.08 ± 0.008^#^	0.073 ± 0.01
P duration (ms)	25.16 ± 2.78	21.49 ± 2.51	19.59 ± 0.5	20.14 ± 0.38
PR interval (ms)	40.81 ± 1.23	41.57 ± 1.79	35.12 ± 0.7^#^	39.50 ± 1.75
R amplitude (mv)	0.47 ± 0.033	0.45 ± 0.021	0.44 ± 0.039	0.46 ± 0.028
JT interval (ms)	30.87 ± 2.84	31.45 ± 0.36	50.44 ± 2.12*^#^	34.37 ± 3.38^$^
T peak T end (ms)	16.3 ± 1.24	15.52 ± 0.75	29.01 ± 0.42*^#^	18.996 ± 3.22^$^
T amplitude (mv)	0.03 ± 0.004	0.03 ± 0.001	0.082 ± 0.002*^#^	0.05 ± 0.01^$^
QT (ms)	55.37 ± 3.94	55.03 ± 0.77	73.31 ± 1.59*^#^	59.35 ± 3.60^$^
QTc (ms)	115.54 ± 8.45	116.16 ± 3.31	188.85 ± 7.33*^#^	139.20 ± 9.93^$^

Untreated hyperthyroidism produced significant tachycardia, decreased PR interval, increased JT duration, T amplitude and duration, and increased QTc duration, as compared to the control group. No significant changes in the other measured ECG parameters. *, #, and $ are significant variances from control, DAPA, and T4 groups in order at *p* ≤ 0.05

### Effect of DAPA on serum biochemical markers

As shown in Table 4, the serum samples of the hyperthyroid (T4) group showed significantly higher concentrations of T3, T4, LDH, and CK-MB and significantly lower concentration of TSH (*p* ≤ 0.05) compared to the control and DAPA groups. On the other hand, DAPA treatment in hyperthyroid rats substantially returned all serum biomarkers to almost their average levels (*p* ≤ 0.05) compared to the T4 group. Of note, no significant change was noticed between the control and DAPA groups in all measured parameters.

**Table 4:** Blood biochemical markers in different groups

Parameter	Control	DAPA	T4	T4 + DAPA
TSH (U/L)	1.16 ± 0.08	1.2 ± 0.05	0.13 ± 0.03*^#^	1 ± 0.05^$^
T_3_ (U/L)	0.43 ± 0.08	0.83 ± 0.06	210.6 ± 20.3*^#^	13.3 ± 1.45^$^
T_4_ (U/L)	24.6 ± 1.2	24.5 ± 0.8	150.1 ± 3*^#^	33.4 ± 1.8*^#$^
LDH (U/L)	1.7 ± 0.08	1.7 ± 0.05	15.9 ± 2*^#^	4.3 ± 0.3^$^
CK-MB (U/L)	0.8 ± 0.08	0.8 ± 0.08	23 ± 0.5*^#^	2.3 ± 0.08*^#$^

*TSH*, thyroid stimulating hormone; *T*_*3*_, threonine; *T*_*4*_, thyroxine; *LDH*, lactate dehydrogenase; *CK*, creatine kinase. Values are mean ± SE, *n* = 6. *, #, and $ are significant variances from control, DAPA, and T4 groups in order at *p* ≤ 0.05

### Effect of DAPA on oxidant/antioxidant biomarker findings

**Fig. 5:**
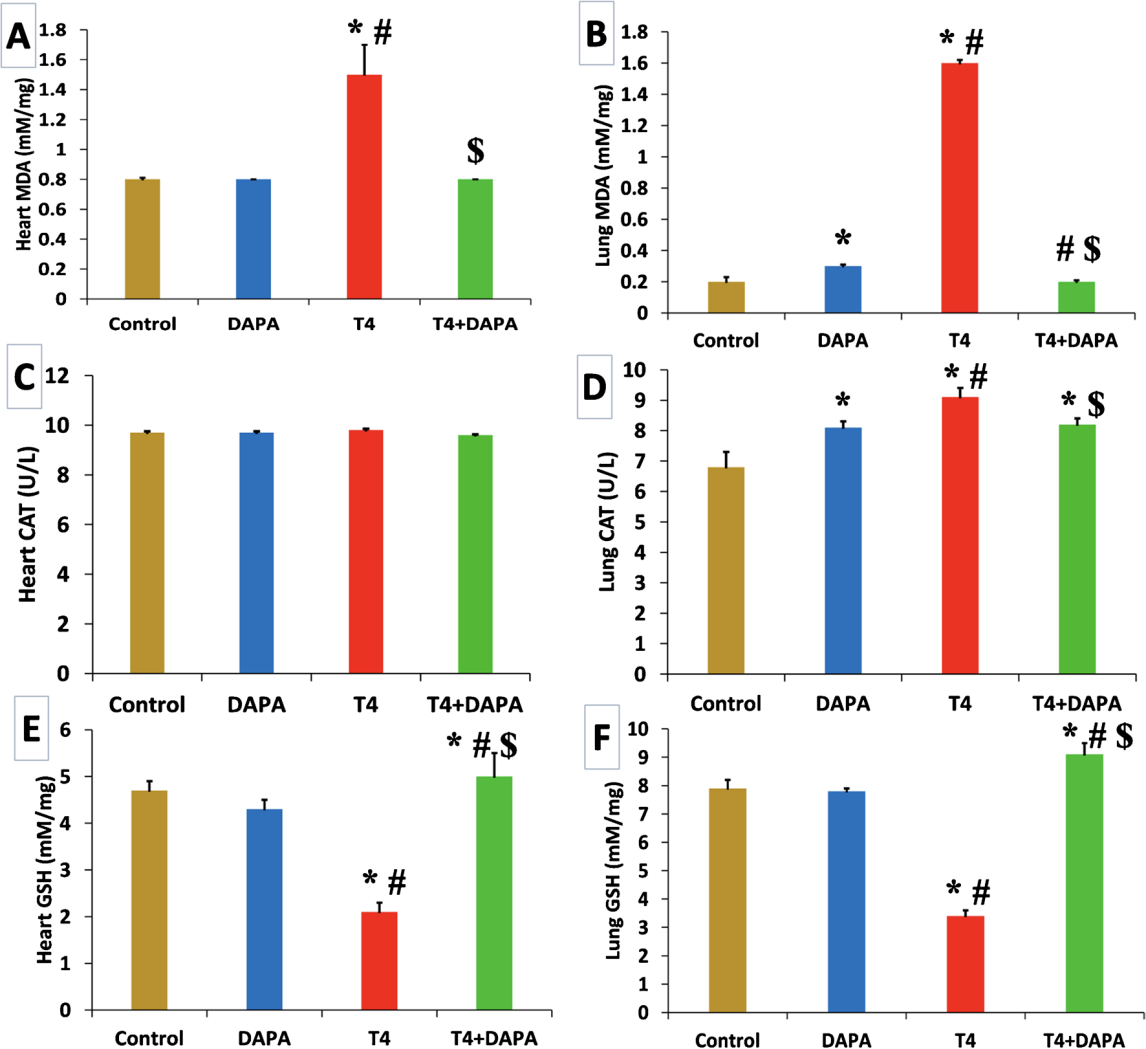
Oxidant/antioxidant biomarkers in different groups. **A** Malondialdehyde (MDA) mM/mg protein content in heart. **B** Malondialdehyde (MDA) mM/mg protein content in lung. **C** Catalase activity U/L in heart. **D** Catalase activity U/L in lung. **E** Reduced glutathione (GSH) mM/mg protein content in heart. **F** Reduced glutathione (GSH) mM/mg protein content in lung. Values are mean ± SE, *n* = 6. *, #, and $ are significant variances from control, DAPA, and T4 groups in order at *p* ≤ 0.05

The concentration of MDA in both organs significantly increased in the T4 group (*p* ≤ 0.05), opposite the control and DAPA groups. However, MDA concentration in both organs was reduced to the average value after DAPA treatment (Fig. 5A, B). The catalase enzyme activity in heart tissue showed a non-significant difference between all study groups (*p* ≤ 0.05). On the other hand, catalase activity in lung tissue significantly raised in the T4 group compared to the control and DAPA groups (*p* ≤ 0.05). Coadministration of DAPA to the hyperthyroid rats resulted in a significant decline in lung catalase activity (*p* ≤ 0.05) compared to the T4 group (Fig. 5C, D). Reduced glutathione concentration significantly decreased in the T4 group of both organs (*p* ≤ 0.05) compared to control and DAPA groups. After DAPA administration, GSH concentration substantially increased (*p* ≤ 0.05) compared to T4 group in both organs (Fig. 5E, F).

### Effect of DAPA on DNA fragmentation percentages in hyperthyroid rats

**Fig. 6:**
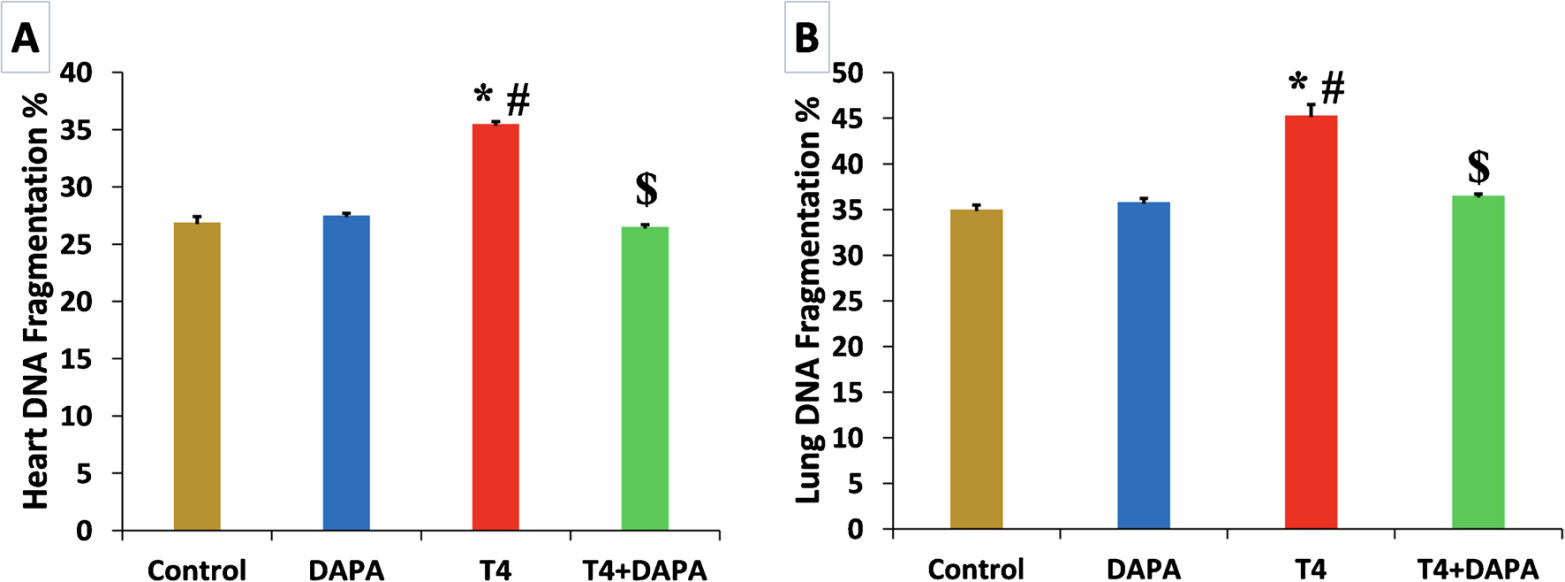
DNA fragmentation % in all groups. **A** Heart DNA fragmentation percentage. **B** Lung DNA fragmentation percentage. Values are mean ± SE, *n* = 6. *, #, and $ are significant variances from control, DAPA, and T4 groups in order at *p* ≤ 0.05

The percentage of DNA fragmentation was considerably elevated (*p* ≤ 0.05) in the T4-treated group in both organs compared to the control and DAPA groups. At the same time, the percentage of DNA fragmentation of both organs returned to average value after DAPA administration with a significant reduction (*p* ≤ 0.05) compared to T4 group (Fig. 6A, B).

### Effect of DAPA on total RNA gene expression in hyperthyroid rats

**Fig. 7:**
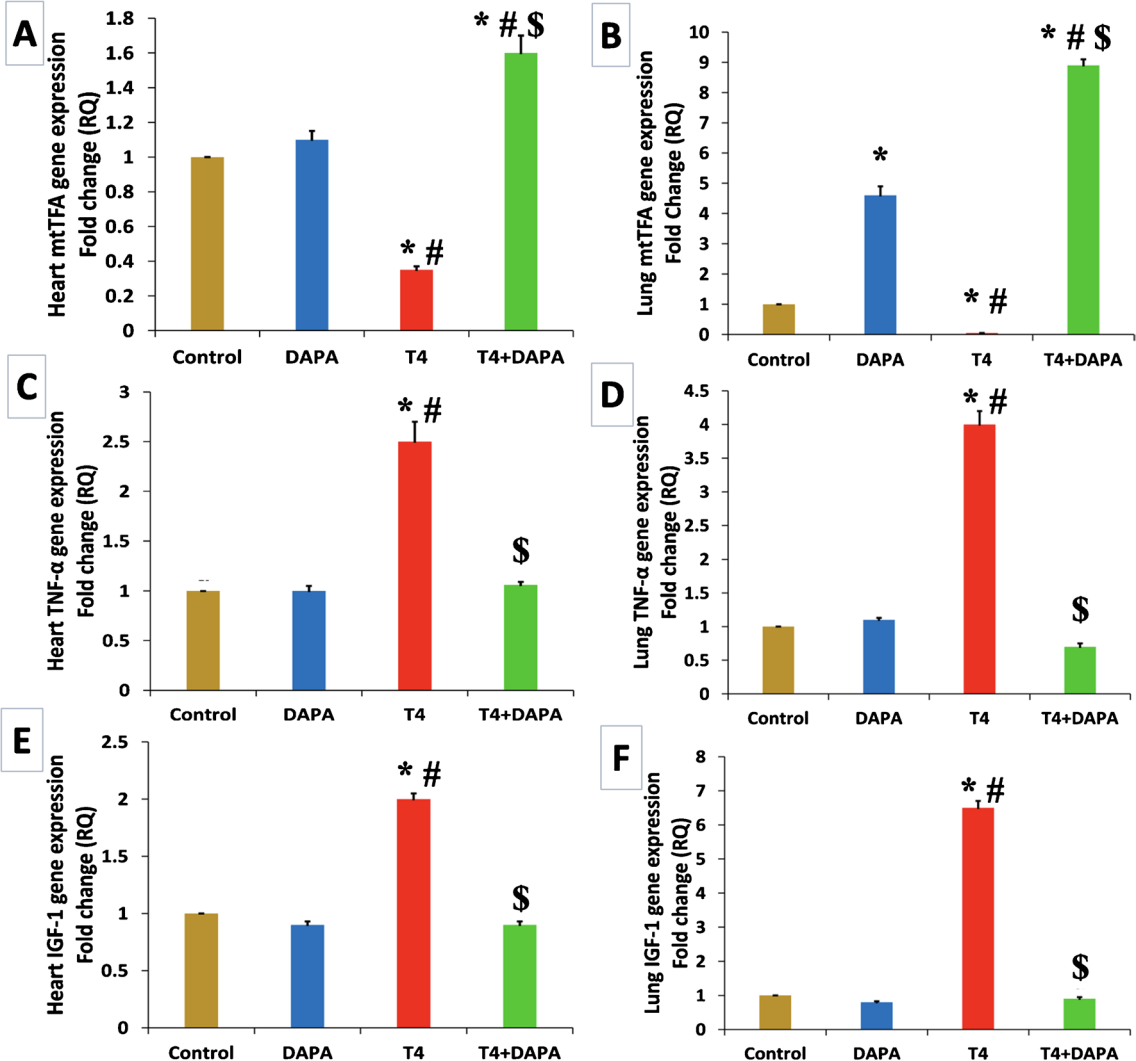
Quantitative RT-PCR of mtTFA, TNF-α, and IGF-1 gene expression in different groups. **A** Evaluation of mtTFA gene expression in the heart. **B** Evaluation of mtTFA gene expression in the lung. **C** Evaluation of TNF-α gene expression in the heart. **D** Evaluation of TNF-α gene expression in the lung. **E** Evaluation of IGF-1 gene expression in the heart. **F** Evaluation of IGF-1 gene expression in the lung. Values are mean ± SE, *n* = 6. *, #, and $ are significant variances from control, DAPA, and T4 groups in order at *p* ≤ 0.05

mtTFA gene expression was significantly regressed (*p* ≤ 0.05) in heart and lung tissues in the hyperthyroid group compared to the control and DAPA groups. After oral DAPA administration, mtTFA expression significantly elevated (*p* ≤ 0.05) to 1.6 and 8.5 folds in heart and lung opposite the control and DAPA groups, respectively (Fig. 7A, B). The expression of the TNF-α gene significantly increased (*p* ≤ 0.05) in the T4-treated groups with significant decrement after medication to restore the average value in both organs (Fig. 7C, D). Gene expression of IGF-1 significantly increased (*p* ≤ 0.05) in T4-treated groups with significant decrement after medication to retrieve to normal value in both organs (Fig. 7E, F).

### Effect of DAPA on the myocardial histoarchitecture in hyperthyroid rats

**Fig. 8:**
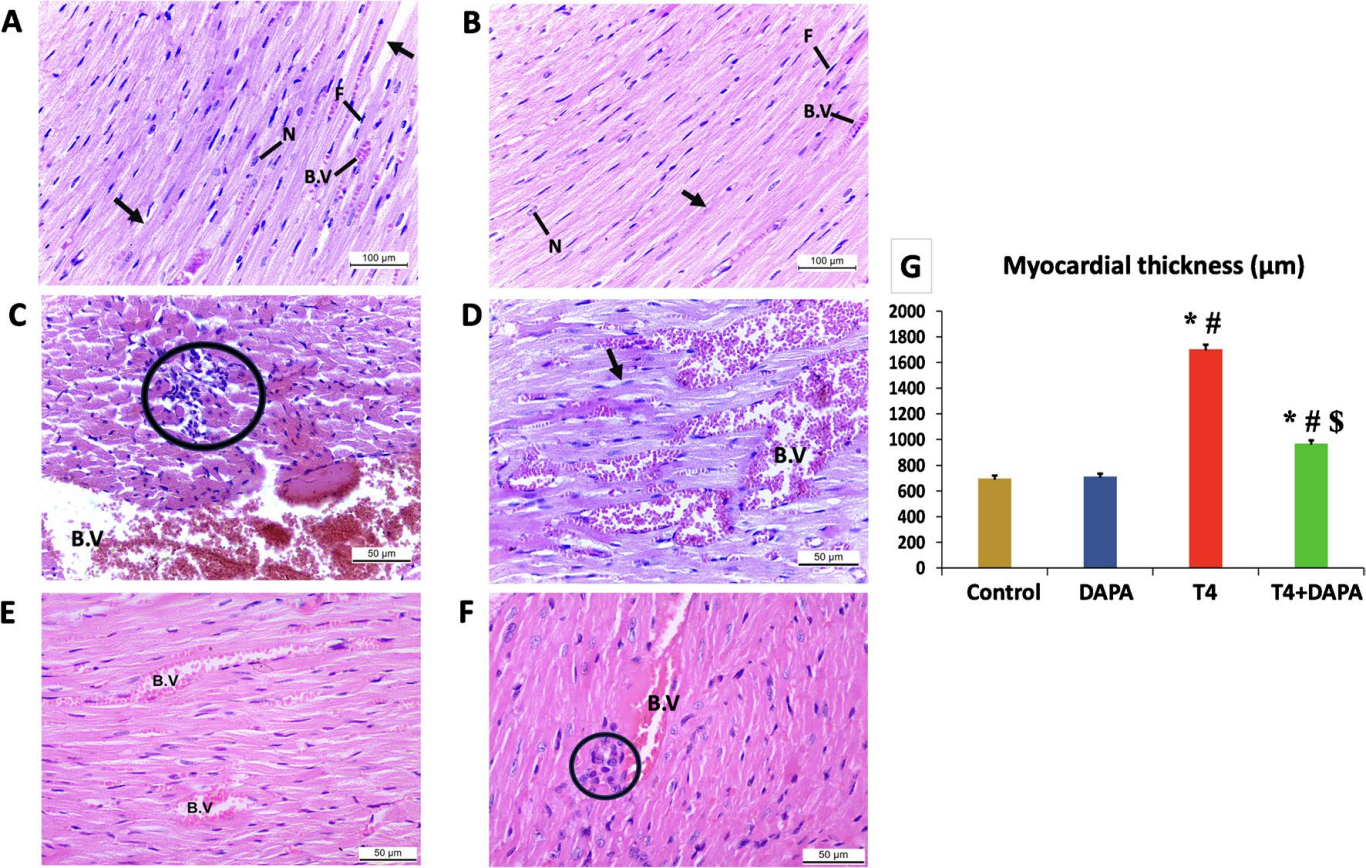
H&E-stained sections from the left ventricle of different research groups. **A**, **B** Control and DAPA groups, respectively, showing typical myocardial architecture, branching striated cardiomyocytes with eosinophilic sarcoplasm (arrows), and central vesicular nuclei (N). Endomysial fibroblasts (F) and narrow caliber capillaries (B.V) intervene between the myocardial fibers. **C**, **D** T4 group showing disturbed myocardial architecture (arrow), inflammatory cellular infiltrates (circle), and marked vascular congestion (B.V). **E**, **F** T4 + DAPA group showing markedly alleviated histopathological signs with mild cellular infiltrates (circle) and less vascular congestion (B.V). **G** Statistical analysis of the myocardial fibers’ thickness in different groups. Values are mean ± SE, *n* = 6. *, #, and $ are significant variances from control, DAPA, and T4 groups in order at *p* ≤ 0.05 (magnification: **A**, **B** × 200; **C**–**F** × 400)

H&E-stained sections from the left ventricle of the control and DAPA groups revealed a normal myocardial architecture. The branching striated cardiomyocytes appeared with eosinophilic sarcoplasm and central vesicular nuclei. Endomysial fibroblasts and narrow caliber capillaries intervened between the myocardial fibers (Fig. 8A, B). Biopsies from the hyperthyroid group showed disturbed myocardial architecture, heavy inflammatory infiltrates, and marked myocardial vascular congestion (Fig. 8C, D). The morphometric measurement of the myocardial fiber diameter in the hyperthyroid group showed a significant rise at *p* ≤ 0.05 compared to the control and DAPA groups. The treatment with DAPA after the induction of hyperthyroidism markedly alleviated the myocardial histopathological signs with mild cellular infiltrates, less vascular congestion (Fig. 8E, F), and a substantial reduction of the myocardial diameter at *p* ≤ 0.05 compared to the hyperthyroid group.

### Effect of DAPA on the myocardial collagen, caspase-3, and TNF-α expression in hyperthyroid rats

**Fig. 9:**
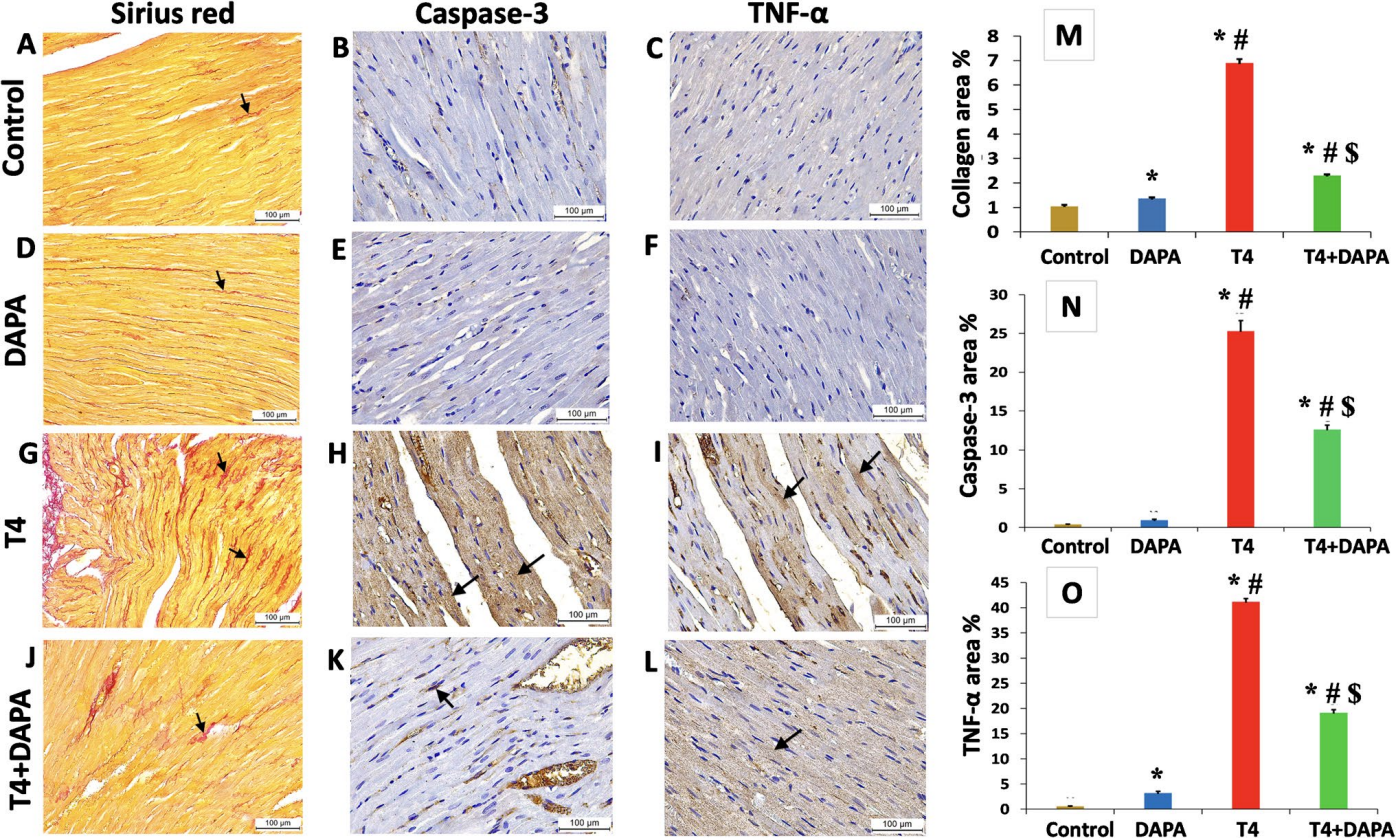
Picrosirius red (**A**, **D**, **G**, and **J**) and immunostained sections with anti-caspase-3 antibodies (**B**, **E**, **H**, and **K**) and anti-TNF-α antibodies (**C**, **F**, **I**, and **L**) from the left ventricle of different research groups. **A**, **D** Control and DAPA groups, respectively, showing delicate red collagen fibers (arrows). **G** T4 group showing thick wavy collagen bundles deposited among the myocardial fibers (arrows). **J** T4 + DAPA group showing less collagen bundles (arrows). **B**, **E** Control and DAPA groups, respectively, showing minimal caspase-3 cytoplasmic expression. (C, F) control and DAPA groups, respectively, showing slight cytoplasmic TNF-α expression. **H**, **I** T4 group showing intense positive cytoplasmic caspase-3 and TNF-α expression, respectively (arrows). **K**, **L** T4 + DAPA group showing less cytoplasmic caspase-3 and TNF-α immunostaining, respectively (arrows) (magnification × 200). **M**–**O** Statistical analysis of the morphometric measurements of collagen, caspase-3, and TNF-α area%, respectively. Values are mean ± SE, *n* = 6. *, #, and $ are significant variances from control, DAPA, and T4 groups in order at *p* ≤ 0.05

Microscopic examination of picrosirius red and immunostained sections from the left ventricle of the control and DAPA groups showed delicate red collagen fibers (Fig. 9A, D, respectively) with a minimal cytoplasmic expression of caspase-3 (Fig. 9B, E, respectively) and TNF-α (Fig. 9C, F, respectively). The hyperthyroid group revealed thick, wavy collagen bundles deposited among the myocardial fibers (Fig. 9G) and intense positive cytoplasmic expression of caspase-3 (Fig. 9H) and TNF-α (Fig. 9I), which showed a considerable increase (*p* ≤ 0.05) opposite the control and DAPA groups. Treatment with DAPA resulted in a significant regression (*p* ≤ 0.05) in the amount of deposited collagen (Fig. 9J), caspase-3 (Fig. 9K), and TNF-α expression (Fig. 9L) compared to the T4 group, yet with a considerable difference opposite the control and DAPA group.

### Effect of DAPA on the lung histoarchitecture in hyperthyroid rats

**Fig. 10:**
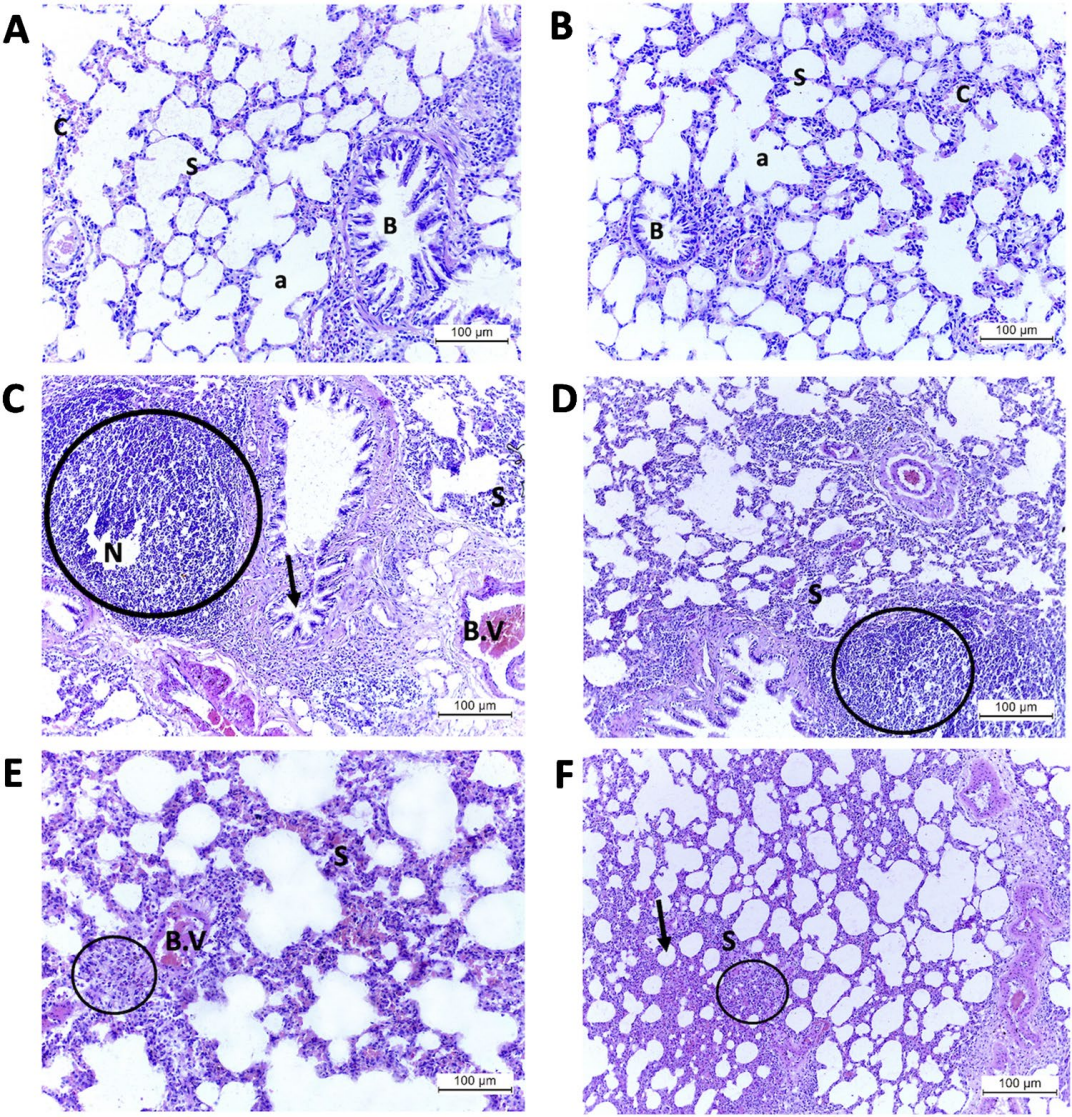
H&E-stained sections from the lung tissue of different research groups. **A**, **B** Control and DAPA groups, respectively, showing many air-filled alveoli and alveolar sacs (a), thin interalveolar septa (s) containing small thin-walled pulmonary capillaries (c), and patent bronchioles with folded mucosal lining (**B**). **C**, **D** T4 group showing thickened interalveolar septa (s), striking inflammatory infiltrates (circle) with areas of necrosis (N), markedly congested pulmonary vessels (B.V), and partially occluded bronchioles (arrow). **E**, **F** T4 + DAPA group showing less vascular engorgement (B.V), milder inflammatory infiltrates (circle), and thickened interalveolar septa (s) with compressed alveolar spaces (arrows) (magnification × 200)

H&E-stained sections of the lung tissue from the control and DAPA groups were identical, showing a typical histologic appearance of lung parenchyma with many air-filled alveoli and alveolar sacs. The interalveolar septa were thin and contained small, thin-walled pulmonary capillaries. Some patent bronchioles were spotted with folded mucosal lining (Fig. 10A, B, respectively). On the other hand, administering LT4 to induce hyperthyroidism led to remarkable histopathologic changes in the hyperthyroid group in the form of noticeably thickened interalveolar septa with striking inflammatory infiltrates and areas of necrosis. The pulmonary vessels showed significant congestion. The bronchioles were compressed to partial occlusion at some points (Fig. 10C, D). After using DAPA as a treatment for the hyperthyroid rats, the lung histoarchitecture acquired a healthier appearance with less vascular engorgement and fewer inflammatory infiltrates. The interalveolar septa were still thickened at some points, resulting in compressed alveolar spaces (Fig. 10E, F). Briefly, the subsequent administration of DAPA after the experimental induction of hyperthyroidism had a tremendous impact on both heart and lung tissues. It restored—at least partially—the normal histologic pattern, which reflects the potential ameliorative effect of DAPA on hyperthyroid-induced cardiopulmonary injury.

### Effect of DAPA on the lung tissue collagen, caspase-3, and TNF-α expression in hyperthyroid rats

**Fig. 11:**
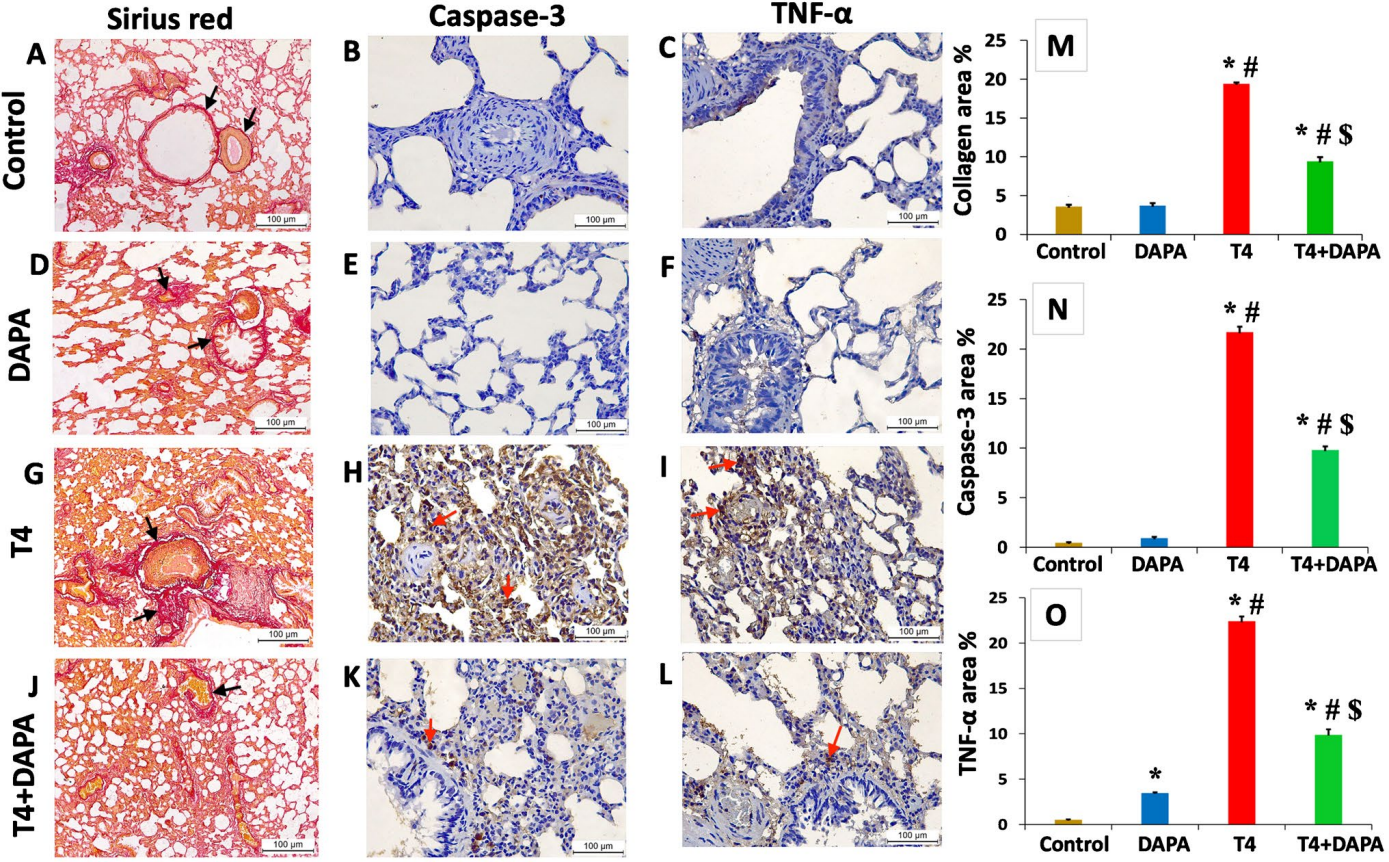
Picrosirius red (**A**, **D**, **G**, and **J**), immunostained sections with anti-caspase-3 (**B**, **E**, **H**, and **K**), and anti-TNF-α (**C**, **F**, **I**, and **L**) antibodies from the lung tissue of different research groups. **A**, **D** Control and DAPA groups, respectively, showing fine red collagen fibers in the parenchyma, adventitia of blood vessels, and bronchioles (arrows). **G** T4 group showing thick collagen bundles (arrows). **J** T4 + DAPA group showing less collagen bundles (arrow). **B**, **E** Control and DAPA groups, respectively, showing minimal caspase-3 cytoplasmic expression. **C**, **F** Control and DAPA groups, respectively, showing weak cytoplasmic TNF-α expression. **H**, **I** T4 group showing strong positive cytoplasmic caspase-3 and TNF-α expression, respectively (arrows). **K**, **L** T4 + DAPA group showing less cytoplasmic caspase-3 and TNF-α reactions, respectively (arrows) (magnification × 200). **M**–**O** Statistical analysis of the morphometric measurements of collagen, caspase-3, and TNF-α area%, respectively. Values are mean ± SE, *n* = 6. *, #, and $ are significant variances from control, DAPA, and T4 groups in order at *p* ≤ 0.05

Picrosirius red and immunostained sections of the lung parenchyma from the control and DAPA groups showed thin red collagen fibers in the parenchyma, the adventitia of blood vessels, and the bronchioles (Fig. 11A, D, respectively), in addition to a weak cytoplasmic expression of caspase-3 (Fig. 11B, E, respectively) and TNF-α (Fig. 11C, F, respectively). The hyperthyroid rats showed a significant increase (*p* ≤ 0.05) in collagen bundles deposition in lung parenchyma (Fig. 11G) besides a strong positive cytoplasmic expression of caspase-3 (Fig. 11H) and TNF-α (Fig. 11I) compared to the control and DAPA groups. The positive effect of administering DAPA to the hyperthyroid rats was proved by the significant decrement (*p* ≤ 0.05) in the amount of deposited collagen (Fig. 11J), caspase-3 (Fig. 11K), and TNF-α expression (Fig. 11L) compared to the T4 group; nevertheless, a considerable difference opposite the control and DAPA group is still noticed.

## Discussion

The hyperthyroidism-induced cardiopulmonary injury was biochemically confirmed in the current study by decreased serum TSH and elevated serum T3, T4, LDH, and CK-MB. Hyperthyroidism induced a significant increase in heart rate and shortened PR interval, with considerable prolongation of QTc and JT intervals, as well as increased amplitude and duration of T wave. In addition, there was a change in the cardiopulmonary redox status, indicating oxidant/antioxidant imbalance in both organs. The analysis of genotoxicity showed that the hyperthyroid rat model’s heart and lungs had a higher percentage of DNA fragmentation than those of the euthyroid animals. Furthermore, compared to euthyroid rats, hyperthyroid rats exhibited a significant rise in the expression of the TNF-α and IGF-1 genes, along with a regression in the expression of the mtTFA gene. Morphologically, hyperthyroidism caused histopathological alterations in the heart and lung tissues in the form of inflammatory infiltrates, areas of necrosis, fibrosis, and vascular congestion. In addition, the morphometric analysis showed a significant increase in the myocardial fiber diameter along with a highly positive cytoplasmic expression of TNF-α and caspase-3 in both organs compared to the control and DAPA groups. Coadministration of DAPA with LT4 effectively restored all serum biomarkers to nearly average levels, improved ECG signs, and reinstated the redox balance. Also, DAPA could improve DNA fragmentation and the expression of mtTFA, TNF-α, and IGF-1 genes in both organs of treated animals. Furthermore, DAPA markedly improved the cardiopulmonary histological alterations, decreased collagen deposition, and reduced the tissue immunoexpression of TNF-α and caspase-3.

LT4 was used in the up-to-date study to establish the rat model with hyperthyroidism. It has been reported that LT4 stimulates thyroid function mainly by preventing the thyroid gland from oxidatively iodinating thyroid hormones [83].

The achievement of inducing hyperthyroidism was demonstrated in our study by a notable rise in serum levels of T3 and T4, as well as a decrease in TSH levels in T4 group; these findings were in line with those of Gluvic and colleagues [24].

DAPA administration to the hyperthyroidism-modeled rats in this study effectively declined T3 and T4 and increased serum TSH levels. This effect could be explained by the reduction of sympathetic activity that SGLT2-I provides. Because SGLT2 is involved in sympathetic nervous system activation, inhibiting it can lower renal afferent nervous activity and suppress central reflex mechanisms that lead to widespread sympathetic activation [61]. Sympathetic nervous system release of norepinephrine from intrathyroidal adrenergic nerve terminals can directly stimulate thyroid hormone secretion. Additionally, it seems that the peripheral metabolism of thyroid hormone and the sympathetic nervous system are related [70]. The former hypothesis was supported by decreased TSH and increased thyroid hormone levels in smokers because of increased sympathetic nervous activity [8].

In coincidence with former studies, the hyperthyroid rat model given LT4 in our study experienced a weight loss that began after the first week of hyperthyroidism induction and reached significant values by the third week of the experiment [38]. Compared to the T4 group, the concurrent SGLT2-I treatment of the hyperthyroid rats considerably reduced their weight loss. Theoretically, after receiving targeted therapy to restore normal hormone levels, patients with thyroid dysfunction should regain their pre-disorder body weight. This is not always the case, though, as multiple studies have demonstrated [31, 43, 57].

The results of ECG recording in the present study showed significant tachycardia with PR shortening in hyperthyroidism (T4) group. Additionally, signs of impaired repolarization were indicated by prolonged JT, QTc, and both T wave amplitude and duration. Opposingly, the T4 + DAPA group did not experience these ECG changes. The ECG alterations linked to hyperthyroidism can be attributed to several pathogenic mechanisms. Increased metabolic rate and sympathetic activity in myocardial cells led to tachycardia and a shorter PR interval [4]. Prolonged JT and QTc intervals were signs of impairment in the repolarization phase of the cardiac action potential [7]. proved that the oxidative stress and abnormal calcium handling by cardiomyocytes resulting from elevated thyroid hormone level were linked to the alteration in the repolarization phase of the cardiac action potential, which was indicated by prolonged JT, QTc intervals, as well as T wave changes. The main reason for the improvement in these results with DAPA treatment is the significant drop in T4 level that was seen in these animals. DAPA dramatically reduced the oxidative and apoptotic effects of thyroid excess at the cellular levels [65]. According to [82], the cardioprotective impact of SGLT2-inhibitors was achieved via the down regulation of sodium-hydrogen antiporter 1, which in turn decreased both intracellular sodium and calcium overloads.

Furthermore, it has been demonstrated that SGLT2 inhibitors affect sympathetic load and lower the risk of heart failure [62]. Remarkably, it was discovered that in a number of pathophysiological stressors, such as heart failure or post-myocardial infarction, cardiomyocytes upregulate the expression of SGLT associated with increased sympathetic activity [14]. The upregulated expression of SGLT in these conditions led to intracellular sodium accumulation and downstream myocardial dysfunction.

Several investigations that attempted to prevent the pathophysiological cascades leading to SGLT overactivity confirmed the causal relationship between SGLT overexpression and cardiomyocyte dysfunction in heart failure. Myocyte morphology and function were preserved as a result of reversing this effect, which was investigated both directly by restricting SGLT activity and indirectly by targeting AMPK activity of cardiomyocytes [63]. The findings of the present study can be explained by these intracellular changes and the potential protection provided by the SGLT inhibitors.

Oxidative stress is considered one of the pathophysiological mechanisms underlying thyrotoxicity. Hypermetabolic state results in accumulation of free radicals both on the systemic and the cellular levels [13]. According to [32], oxidative stress increased the generation of reactive oxygen species (ROS) in diabetes-induced cardiomyopathy, which resulted in cardiac apoptosis.

Our results revealed a substantial rise in MDA level in the heart and lung tissues of hyperthyroid rats opposite the controls, a significant decline in the GSH profile in both organs, and an increase in the CAT levels in the lungs alone. This finding was in line with former studies which reported a significant rise of CAT, SOD, and MDA levels in the liver, kidney, brain, and myocardial tissues of hyperthyroid rats compared to the controls [49, 60]. On the contrary, [45] observed a marked decline in myocardial tissue catalase and SOD in rat model of thyrotoxicosis. According to Costilla et al., a hyperthyroid condition raises ROS, which triggers the transcription of antioxidant enzymes. That could indicate that the oxidative stress is not adequately compensated for [17]. Lipid peroxidation is induced in various rat tissues, including the heart, by LT4-induced hyperthyroidism [47]. Polyunsaturated fatty acid peroxidation results in the production of MDA. It can bind to RNA and DNA, damaging cells [36]. Significant oxidative reactions occur when ROS interacts with deoxyribose and nitrogenous bases in DNA. Mutations, apoptosis, necrosis, carcinogenesis, and hereditary illnesses can result from this. Nucleosomes, which are essential for the organization of DNA within chromosomes, rupture, leading to DNA fragmentation. This causes issues with the compaction and coiling of DNA within chromatin. The control of gene transcription is significantly influenced by chromatin [11]. The study’s hyperthyroid animals also displayed higher percentages of DNA fragmentation in their lung and cardiac tissues, confirming the production of hydroxyl radicals, the main species that damage DNA more severely [33]. Also, DNA damage is documented in our study by the downregulation of the mtTFA gene. Nuclear genes encode the mitochondrial protein known as mtFTA, which is moved from the cytoplasm to the mitochondria. Mitochondrial DNA must be maintained, expressed, and delivered. mtTFA also controls mitochondrial DNA transcription, stability, repair, and replication [69]. Oxygen radical stress oxidatively damages purine and pyrimidine bases and directly damages DNA, primarily through strand excision. This process can produce a variety of products and begins with the radical-induced abstraction of a proton from any position on the deoxyribose [55]. Overexposure to ROS decreased the potential of the mitochondrial membrane, which resulted in immunotoxicity and apoptotic cell death due to an imbalance in immune redox [9]. This is supported in the present study by the immunohistochemical results, which showed elevated levels of apoptotic protein caspase-3 in hyperthyroid rats’ heart and lung tissues. Similar results were observed in previous work [60].

Our study showed a significantly increased cardiopulmonary gene expression of IGF-1 in the hyperthyroid group, while a significant decline was noticed after DAPA treatment. The previous finding was in line with previous studies which reported elevated IGF-1 levels with goiter in males who have thyroid nodules, in females who have low TSH levels, and in patients with primary and central hypothyroidism after T4 replacement [64, 77]. It has been previously mentioned that hyperactivation of insulin/IGF-1 signaling in diabetic patients favors cell survival via inhibiting P1-3 K and SIRT1, which provide a downstream activation of the p53-p21 signaling pathway. In this regard, SGLT2 inhibitors might prevent cells’ exposure to oxidative stress and promote cellular repair via up-regulating energy deprivation sensors such as AMPK and SIRT1, as well as via inhibition of insulin/IGF-1 signaling [28].

In the current work, treatment with dapagliflozin exerted a cardiopulmonary antioxidant effect via ameliorating oxidative stress and boosting the antioxidant capacity in heart and lung tissues. Dapagliflozin exerted its antioxidant properties by reducing oxidative stress and boosting cardiomyocytes’ antioxidant potential [50]. There is minimal evidence of SGLT2 expression, while the cardiomyocytes express the SGLT1 isoform [29]. Therefore, dapagliflozin’s antioxidant effect was attributed by Xing and colleagues using in vitro experiments, to its direct inhibition of ROS production [81].

In the present work, hyperthyroidism-induced cardiopulmonary injury was evidenced biochemically by elevated serum levels of LDH and CK-MB. In many diseases, changes in isoenzymes and plasma or serum enzymes can serve as helpful markers of tissue damage. Increases in enzymes are typically associated with their leakage from injured cells. Elevated LDH level is a nonspecific finding and is observed in various hematological and neoplastic disorders, as well as in diseases of the heart, liver, lungs, skeletal muscles, and kidneys [39]. Elevated levels of LDH and CK-MB are positively correlated with ischemic myocardial injury in clinical practice. Both markers are released into the bloodstream during myocardial injury [16].

Furthermore, the hyperthyroidism-related myocardial injury was proven histologically in our study by disturbed histoarchitecture, increased collagen deposition, severe inflammation, vascular congestion, and increased diameter of the myocardial fibers. These results were the same as those of [35, 60]. On the pulmonary side, and in line with [30, 78], hyperthyroidism led to remarkable histopathologic changes in the lung tissues in the form of thickened interalveolar septa with striking inflammatory infiltrates, areas of necrosis, congested pulmonary vessels, and compressed bronchioles that were partially blocked at some points.

Interestingly, a previous study reported the same pulmonary histopathological changes in a rat model of hypothyroidism [26], which could be attributed to the evoked proinflammatory markers (mainly TNF-α and IL-6) and the deranged redox status accompanying the disturbed euthyroid state.

Moreover, cardiac hypertrophy development is influenced by hyperthyroidism. Two important pathways in cardiac hypertrophy are thought to be endoplasmic reticulum stress and transient receptor potential canonical channels [10]. Furthermore, β-adrenergic receptors, phospholamban, myosin heavy chains, and other proteins in the cardiac myocyte are all regulated by thyroid hormones, which can cause cardiac hypertrophy [76]. The reversibility of cardiomyopathy resulting from hyperthyroidism is contingent upon the timing and the proper management of the hyperthyroid condition. Patients with hyperthyroidism may, therefore, benefit from the administration of cardioprotective agents [51]. In our study, the substantial reduction in CK-MB and LDH levels, as well as the attenuation of cardiopulmonary histopathological changes, demonstrated the protective action of SGLT2-I. Empagliflozin has been shown by Andreadou and colleagues [5] to have a cardioprotective potential against myocardial ischemia injury in animals given a Western diet. Furthermore, in type 2 diabetic mice, DAPA decreased cardiomyopathy [72].

Increased tissue and plasma concentrations of inflammatory cytokines are linked to inflammation [21]. IL-6, IL-8, and TNF-α are among the cytokines that are elevated in hyperthyroidism; this increment might be the consequence of the long-term effects of elevated thyroid hormones [22]. Earlier research indicated that LT4-induced hyperthyroidism was linked to an elevation in TNF-α [44, 60], which aligns with our immunohistochemical results that showed a robust positive cytoplasmic expression of TNF-α in the hyperthyroid rats compared to the euthyroid controls. A rise in inflammatory cytokines like TNF-α brought on by NF-кB activation resulted in cardiac complications [79]. SGLT2-I coadministration to hyperthyroid rats significantly decreased TNF-α levels. Per our results, Eldesoqui et al. reported that TNF-α-antagonism was linked to a reduction in myocardial inflammation and fibrosis and that it improved diabetic cardiomyopathy that was experimentally induced [18].

## Conclusion

Our present study demonstrated that the deranged oxidant/antioxidant balance accompanied by the evoked proinflammatory and proapoptotic markers is the chief cause of hyperthyroidism-induced cardiopulmonary injury. Combining DAPA with LT4 could effectively regulate inflammation and apoptosis by downregulating TNF-α and caspase-3, combating genotoxicity, and reinstating the redox balance in heart and lung tissues. Further, DAPA exhibited a sympatholytic effect, documented by the improved hyperthyroid-related ECG changes, reflecting better cardiac function. Hence, our study introduces DAPA as a putative therapeutic candidate with cardiopulmonary protective potential in patients with hyperthyroidism. Further clinical and deep molecular studies are required before transposing to humans.

## Limitations of the study

Our study could be improved by further molecular studies encompassing the effect of DAPA on autophagy in myocardial cells and pulmonary tissue and electron microscopic studies to locate the cellular ultrastructural targets that open the door for future research. In addition, we suggest performing serial ECG recordings throughout the experiment to track early changes in cardiac function by the effect of levothyroxine and the putative protective role of DAPA.

## Data Availability

No datasets were generated or analyzed during the current study.
